# Correlation-Assisted Pixel Array for Direct Time of Flight

**DOI:** 10.3390/s24165380

**Published:** 2024-08-20

**Authors:** Ayman Morsy, Maarten Kuijk

**Affiliations:** Department of Electronics and Informatics, Vrije Universiteit Brussel, Pleinlaan 2, 1050 Brussels, Belgium; maarten.kuijk@vub.be

**Keywords:** direct time of flight (dToF), time of flight (ToF), single-photon avalanche diode (SPAD), light detection and ranging (LiDAR), depth sensing, 3D imaging

## Abstract

Time of flight is promising technology in machine vision and sensing, with an emerging need for low power consumption, a high image resolution, and reliable operation in high ambient light conditions. Therefore, we propose a novel direct time-of-flight pixel using the single-photon avalanche diode (SPAD) sensor, with an in-pixel averaging method to suppress ambient light and detect the laser pulse arrival time. The system utilizes two orthogonal sinusoidal signals applied to the pixel as inputs, which are synchronized with a pulsed laser source. The detected signal phase indicates the arrival time. To evaluate the proposed system’s potential, we developed analytical and statistical models for assessing the phase error and precision of the arrival time under varying ambient light levels. The pixel simulation showed that the phase precision is less than 1% of the detection range when the ambient-to-signal ratio is 120. A proof-of-concept pixel array prototype was fabricated and characterized to validate the system’s performance. The pixel consumed, on average, 40 μW of power in operation with ambient light. The results demonstrate that the system can operate effectively under varying ambient light conditions and its potential for customization based on specific application requirements. This paper concludes by discussing the system’s performance relative to the existing direct time-of-flight technologies, identifying their strengths and limitations.

## 1. Introduction

Time of flight (ToF) is a method for detecting the distance between an object and a sensor using a modulated light source with a certain wavelength. The time taken by light to pass through a medium corresponds to the object’s distance from the sensor. The method of detecting the arrival time of the light source determines the technology used. However, the environment often contains other light sources of the same wavelength, such as sunlight, LED light, and lamp light, collectively referred to as ambient light. The optical sensor detects any light source in the environment, including the ToF light source. To accurately extract the ToF signal, various methods have been developed to cancel out ambient light.

One approach is using indirect time of flight (iToF). In iToF, a modulated light source illuminates a scene synchronized with a photonic mixer device shutter. The phase difference between the modulated shutter and the reflected light determines the time of flight. The light source can be a pulsed emitter or a continuous wave emitter with sinusoidal or square modulation [1]. A widely used iToF scheme is the amplitude-modulated continuous-wave (AMCW) method, due to its robustness and scalability [2]. In this operation, a four-phase time gate is measured to detect the phase difference using 50% duty cycle modulated light. However, ambient light shot noise can limit pixel operation. Therefore, several publications (VGA and higher image resolution) have demonstrated an operational range of four meters under limited ambient light and a high modulation frequency [3,4,5]. Additionally, a high irradiance level can saturate the limited well capacitance, particularly in a small pixel pitch. To address pixel saturation, a binning technique has been reported [3,6]. Although this technique allows for high-resolution systems, it is limited in range due to ambient light shot noise.

Various schemes have been developed to suppress ambient light. One approach is to utilize a hybrid ToF (hToF) pixel with multiple tapes [7]. In this pixel operation, a pulsed laser source is synchronized with multiple time-gated windows to determine the laser arrival window overlap. Each frame is divided into four sub-frames, each divided into four sequential windows in time. The detection range is determined by shifting the time windows from one sub-frame to the next. The hToF method improves ambient light suppression and maximizes the detected laser pulses, avoiding full well capacitance saturation and reducing ambient shot noise. The hToF pixel has been demonstrated outdoors with a maximum nonlinearity of 4% over a 10 m detection range, with precision of 1.6% [8]. To achieve a longer detection range over one frame, an eight-tap pixel has been implemented, reducing nonlinearity to 0.6% over an 11.5 m range with precision of 1.4% [9]. Another approach is a multi-range four-tap pixel array, for which each frame is divided into three sub-frames (near, middle, and far range of detection) [10]. The pixel showed outdoor operation nonlinearity of 1.5% over 20 m with precision of 1.3%.

Due to limited signal reflection from distant objects, iToF requires a longer integration time, leading to a limited frame rate and potential full well saturation. A solution to low sensitivity over far objects is using a single-photon avalanche diode (SPAD) sensor, offering high sensitivity, low timing jitter of a few hundred picoseconds, and high response time. The SPAD was integrated into the iToF circuit using a correlated pulsed laser source with an internal clock, employing high-frequency modulation to achieve high precision [11]. According to the clock signal, the pixel integrates the detected photon by counting up and down over a capacitor. This system exploits the uniform arrival time of ambient light. Thus, the integrated voltages from ambient light triggers are canceled out for a sufficient integration time. However, this operation is constrained by the capacitor saturation over strong irradiance, limiting the pixel’s dynamic range. Additionally, the detection range is restricted to 1.5 m.

Direct ToF (dToF) is another approach in which photons’ arrival time is directly recorded, and a histogram is generated from the collected data. This method employs a short-pulsed laser source paired with an avalanche detector, with the arrival time being converted into either digital or analog values [1]. Time-to-digital converters (TDCs) are the most commonly used technique in dToF systems, with various architectures such as ring oscillators [12], a multi-phase clock [13], and a Vernier delay loop per pixel [14]. Recent advancements in TDC architectures and calibration techniques aim to improve the system linearity [15]. Multiple laser cycles are detected, with the time bin resolution depending on the TDC architecture and detection range. The ambient light appears in the histogram as a uniform offset with fluctuation due to shot noise, while the detected laser pulse bins have higher counts. SPAD-based dToF systems are popular due to the advantageous characteristics of the SPAD, enabling dToF operation over ranges from a few meters to several kilometers, depending on the laser power used. The accuracy and precision in dToF systems depend on the TDC architecture and resolution, which consequently affects the data rate transferred on-chip. This data rate can range from a few gigabits to several terabits per second. A higher image resolution increases the data rate, leading to higher power consumption and limiting the achievable frame rate. Additionally, data management poses a significant challenge in dToF systems, increasing the system complexity. Various approaches have been proposed to address data management issues by incorporating dedicated on-chip units to store and process some of the data before they are transferred off-chip. One such approach involves developing an in-pixel histogram, where the detected peak is processed in-pixel after background count compensation [16]. However, the pixel size is 114 × 54 μm${\;}^{2}$, developed using a 40 nm front-side illumination (FSI) technology, limiting its scalability to a high-resolution pixel array. Another limitation in dToF pixels is pile-up distortion, which arises from SPAD deadtime and the limited TDC conversion rate, leading to inaccurate peak detection [17].

This paper proposes an SPAD-based pixel array capable of in-pixel ambient light suppression, characterized by low power consumption and a simple pixel structure. The design facilitates scalability to a high image resolution. The pixel’s working principle and schematic are presented in Section 2. In Section 3, we develop an analytical model for the pixel, which is subsequently verified using a statistical model in Section 4. A 32 × 32 pixel array is fabricated and tested as a proof of concept for pixel operation. Finally, we discuss the pixel’s performance and compare it with other state-of-the-art pixels in Section 6.

## 2. Correlation-Assisted Direct Time-of-Flight Pixel Principle

**Figure 1:**
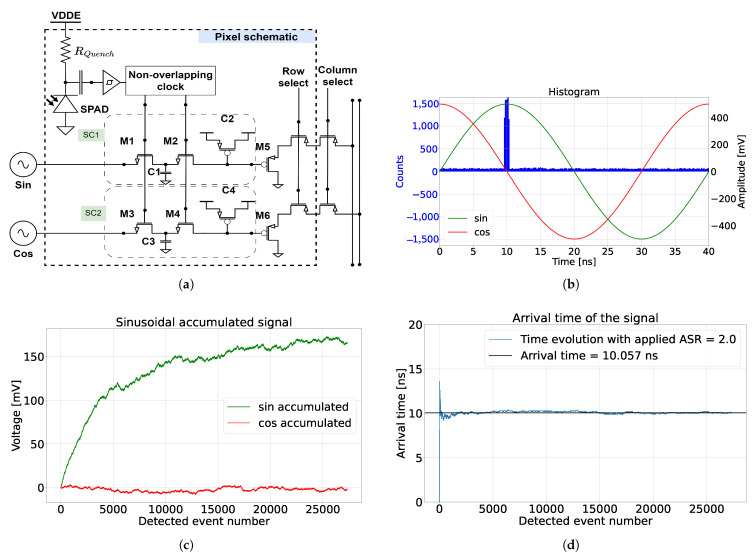
CA-dToF pixel schematic and simulation, where (**a**) is the pixel schematic and (**b**) is the histogram from the detected events. On the right side is the sinusoidal signals applied to the CA-dToF pixel, while (**c**) is the voltage evolution of the analog channels SC1 and SC2 and (**d**) is the calculated arrival time, with ASR = 2.

To introduce the operation principle of a correlation-assisted direct time-of-flight (CA-dToF) pixel, we first introduce the pixel schematic in Figure 1a. A laser pulse is synchronized with two orthogonal sinusoidal signals, where the two signals are applied to the pixel as inputs. The pixel utilizes a passively quenched SPAD with a resistor $R_{Quench}$ to detect the photon arrival time. The SPAD triggers may originate from the reflected laser pulse, ambient light, or dark count events. When the SPAD is triggered, a non-overlapping clock is generated, driving the two switched capacitor channels ($M_{1}$ and $M_{2}$ for SC1 and $M_{3}$ and $M_{4}$ for SC2). Each channel evolves via the following equation:

(1) $$\begin{array}{c} V_{i}=\frac{V_{m}}{n_{av}}+\left(1-\frac{1}{n_{av}}\right)V_{i-1}, \end{array}$$

where $V_{m}$ is the sampled voltage of the applied sinusoidal signal and $n_{av}=\frac{C_{1}+C_{2}}{C_{1}}=\frac{C_{3}+C_{4}}{C_{3}}$ is defined as the integration length. Equation (1), known as the exponential weighted moving average (EWMA), is explained in detail in Appendix A. The system is averaging the detected voltage over multiple iterations, with a slow reduction of the weight $1/n_{av}$ for a high integration length value $n_{av}$, meaning that $C_{1},C_{3}<<C_{2},C_{4}$.

The pixel operation involves converting the time-of-arrival information into a voltage. Therefore, it is a form of time-to-amplitude conversion (TAC). To provide intuitive understanding of the system’s operation, a simulation was conducted and presented in Figure 1, where $n_{av}=4000$. The simulation presents top-hat laser pulses of 1.7 ns full-width half maximum (FWHM) with an arrival time of 10 ns to a passively quenched SPAD sensor with 5 ns of deadtime. Sinusoidal signals with a 40 ns period were applied for 1000 cycles over the entire simulation period. A histogram of the simulation and the applied signal is depicted in Figure 1b. The ambient light and dark count events were double the applied laser pulse photons, making the ambient-to-signal ratio (ASR) equal two on average. All the incident events before detection followed a Poisson distribution. The simulation result in Figure 1c shows the evolution of the accumulated voltage across the switched capacitor channels SC1 and SC2. The phase evolution of the detected events, which represents the laser pulse arrival time, is calculated and presented in Figure 1d. Notably, the detected phase reached equilibrium after 2000 incident events, while the accumulated voltages required nine times more photons to reach equilibrium. An output reaches equilibrium when the output does not vary significantly anymore, and it oscillates around a certain value. The voltage evolution behavior is known as the inertia effect of the system, which is explained in Appendix A.3. Intuitively, this phenomenon is directly related to the integration length $n_{av}$, where the bigger $n_{av}$ is, the more detected events are needed for the switched capacitor voltage to reach equilibrium. However, as presented in Figure 1d, the inertia effect does not affect the phase information, because the phase is the ratio of the two voltages and not the absolute voltages. As the pixel is exponentially averaging the detected amplitude of the sinusoidal signal, the system does not suffer from saturation or overflow for high input photon flux, as long as the SPAD is not in saturation.

Four key parameters influence pixel operation, which are (1) the number of incident laser and ambient photons, (2) the integration length {$n_{av}$}, (3) the laser pulse width {*a*}, and (4) the applied sinusoidal signal amplitude {*C*}. To examine pixel behavior, an analytical model of the pixel is provided in Section 3, with emphasis on developing useful tools to extract more information about the environment. The analytical model was validated through simulations under varying conditions, as described in Section 4. The experimental results are detailed in Section 5.

## 3. CA-dToF Analytical Model

### 3.1. Ambient Light Influence

The ambient light is not correlated with the arrival time, quantified by a rate {*A*} (#/ns). Consequently, the arrival time probability distribution is uniformly spread across the integration time. This presumption encompasses light sources such as sunlight and LED sources emitting photons without temporal correlation. However, the assumption excludes light sources exhibiting a specific frequency. For a light source to be categorized as ambient, it must lack correlation with the used active light source, resulting in a uniform distribution in arrival time throughout the integration time. Hence, light sources with harmonics which do not resonate with the active light source remain classified as ambient light, such as indoor lighting and vehicle headlights.

**Figure 2:**
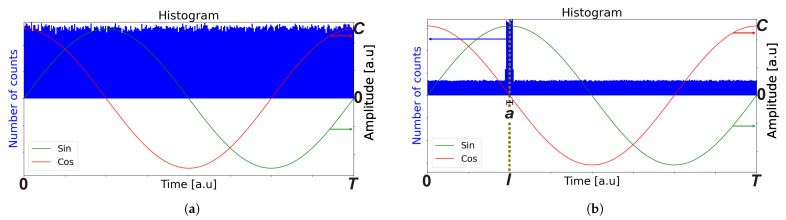
(**a**) Histogram of accumulated ambient light over a period {*T*} for a certain integration time. (**b**) Histogram of ambient light and laser pulses with an FWHM {*a*} detected with an arrival time {*l*} over a period {*T*}, along with ambient light that is uniformly distributed over the integration time.

The system utilizes two orthogonal sinusoidal signals with a period *T*, where each photon detected at time *t* generates a corresponding voltage such that $I\left(t\right)=C\cdot \sin\left(\frac{2\pi }{T}t\right)\left[V\right]$ and $Q\left(t\right)=C\cdot \cos\left(\frac{2\pi }{T}t\right)\left[V\right]$, where {*C*} is the applied signal amplitude shown in Figure 2a. The detected voltages $\hat{I}$ and $\hat{Q}$ are independent of each other, indicating that the measurement of one signal does not influence the measurement of the other. Therefore, they can be considered independent variables.

The expected values of the variables $\hat{I}$ and $\hat{Q}$ over an appropriate integration time can be calculated by integrating the random variables over a period {*T*} with a uniform probability distribution f(t)dt=(A·dt)/(A·T)=dt/T. The results are

(2) $$E\left[\hat{I}\right]=E\left[\hat{Q}\right]=0.$$

The variance of the random variables $\hat{I}$ and $\hat{Q}$ over an appropriate integration time is

(3) $$\begin{array}{c} \sigma_{\hat{I}}^{2}=\sigma_{\hat{Q}}^{2}=\frac{L^{2}}{\left(2n_{av}-1\right)}\frac{C^{2}}{2}\;1\leq L\leq 3, \end{array}$$

where *L* is the control width, which represents the model tolerance [18]. The physical interpretation of the results can be understood by the fact that the applied sinusoidal signals exhibit intrinsic symmetry around a certain voltage (in this case, $V_{offset}=0$). Consequently, if the probability of detection is equal over the signal period, then the voltage’s expected value converges to zero volts, enabling the system to suppress ambient light. The voltage variance, however, is related to the ambient light shot noise. We will refer to the standard deviation of the detected voltages ($\sigma_{\hat{I}}$ and $\sigma_{\hat{Q}}$) as the amplitude precision.

### 3.2. Active Light Influence

To simplify the analysis, we considered top-hat laser pulses with a (FWHM) {*a*} centered around time {*l*} which were periodically received over a period {*T*}. When ambient light and laser pulses are applied, the accumulated photons over the integration time are depicted in Figure 2b.

### 3.3. Expected Value

In the presence of laser pulses, the expected values of the sinusoidal signals $\hat{I}$ and $\hat{Q}$ over an appropriate integration time can be calculated in a similar manner to that in the previous section, with the appropriate probability distribution of $f\left(t\right)=\frac{A}{S\cdot a+A\cdot T}$ when there is ambient light and $f\left(t\right)=\frac{S+A}{S\cdot a+A\cdot T}$ when there is a laser pulse, where {*S*} is the laser photon arrival rate (#/ns). The expected values are

(4) $$\begin{array}{c} E\left[\hat{I}\right]\left(l\right)=\frac{S\cdot C}{S\cdot a+A\cdot T}\frac{T}{\pi }\left(\sin\left(\frac{2\pi }{T}l\right)\sin\left(\frac{\pi }{T}a\right)\right),\\ E\left[\hat{Q}\right]\left(l\right)=\frac{S\cdot C}{S\cdot a+A\cdot T}\frac{T}{\pi }\left(\cos\left(\frac{2\pi }{T}l\right)\sin\left(\frac{\pi }{T}a\right)\right). \end{array}$$

In Equation (4), we can observe that the norm of $\hat{I}$ and $\hat{Q}$ is invariant:

(5) $$\begin{array}{c} \hat{C}=\sqrt{E\left[\hat{I}\right]{\left(l\right)}^{2}+E\left[\hat{Q}\right]{\left(l\right)}^{2}}=\frac{S\cdot C}{S\cdot a+A\cdot T}\frac{T}{\pi }\sin\left(\frac{\pi }{T}a\right), \end{array}$$

which is referred to as the system’s confidence. For a short laser pulse {*a*}, and using the small angle approximation, the amplitude $\hat{C}$ is approximated to be $\hat{C}\approx \frac{S\cdot a}{S\cdot a+A\cdot T}C=\frac{1}{1+ASR}C,$ where $ASR=\frac{A\cdot T}{S\cdot a}$. Consequently, when the system is at equilibrium, ASR is approximately

(6) $$\begin{array}{c} ASR\approx \frac{C-\hat{C}}{\hat{C}}. \end{array}$$

**Figure 3:**
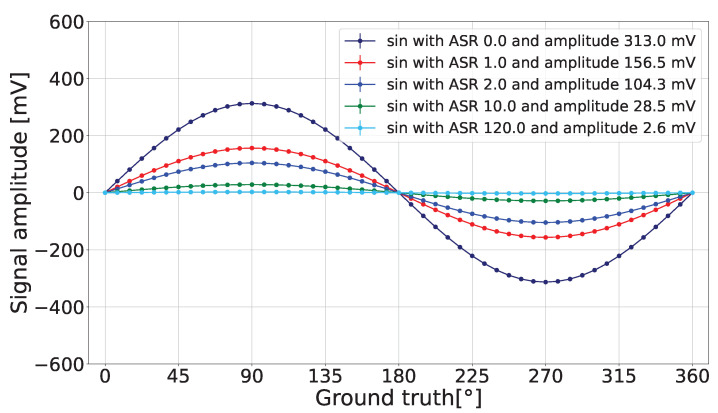
Reduction in the detected sine’s amplitude for different ASR values when a=4.25%·T and C=274.6 mV.

The detected amplitude reduction is visually presented in Figure 3.

For the small angle approximation deviation to be ≤1%, Equation (6) is valid when the pulse width is restricted by the following relation:

(7) $$\begin{array}{c} a\leq 7.8\%\cdot T. \end{array}$$

For example, if the period is T=40 ns, then Equation (6) is valid when a≤3.12 ns.

### 3.4. Variance

The variance in $\hat{I}$ and $\hat{Q}$ can be calculated with the same probability distribution shown in Section 3.3 and Equation (A5). The variance equations are

(8) $$\begin{array}{ccc} \sigma_{\hat{I}}{\left(l\right)}^{2} & = & \frac{L^{2}}{\left(2n_{av}-1\right)}[\frac{A\cdot T\cdot C^{2}}{2(S\cdot a+A\cdot T)}+\frac{S\cdot C^{2}}{S\cdot a+A\cdot T}\frac{T}{2\pi }\left(\frac{\pi a}{T}-\frac{1}{2}\left(\cos\left(\frac{4\pi }{T}l\right)\sin\left(\frac{2\pi }{T}a\right)\right)\right)\\ & - & {\left(\frac{S\cdot C}{S\cdot a+A\cdot T}\frac{T}{\pi }\left(\sin\left(\frac{2\pi }{T}l\right)\sin\left(\frac{\pi }{T}a\right)\right)\right)}^{2}], \end{array}$$

(9) $$\begin{array}{ccc} \sigma_{\hat{Q}}{\left(l\right)}^{2} & = & \frac{L^{2}}{\left(2n_{av}-1\right)}[\frac{A\cdot T\cdot C^{2}}{2(S\cdot a+A\cdot T)}+\frac{S\cdot C^{2}}{S\cdot a+A\cdot T}\frac{T}{2\pi }\left(\frac{\pi a}{T}+\frac{1}{2}\left(\cos\left(\frac{4\pi }{T}l\right)\sin\left(\frac{2\pi }{T}a\right)\right)\right)\\ & - & {\left(\frac{S\cdot C}{S\cdot a+A\cdot T}\frac{T}{\pi }\left(\cos\left(\frac{2\pi }{T}l\right)\sin\left(\frac{\pi }{T}a\right)\right)\right)}^{2}]. \end{array}$$

The model demonstrates the relationship between the amplitude precision of the two signals $\sigma_{\hat{I}}$ and $\sigma_{\hat{Q}}$ and the ambient light rate {*A*}, the signal light rate {*S*}, the pulse width {*a*}, and the signal period {*T*}. It consists of two main terms:

- The ambient shot noise term ($\frac{A\cdot T\cdot C^{2}}{2(S\cdot a+A\cdot T)}$), which is directly related to the ambient light;
- The combination of laser and ambient shot noise, which is influenced by the laser pulse width and the ASR.

Notably, through Equations (8) and (9), the amplitude precision can be improved by increasing the integration length {$n_{av}$}. Consequently, the system allows counteracting high ambient light effects by adjusting its integration length {$n_{av}$} at the cost of increasing the inertia effect.

Section 3.5 is dedicated to providing an intuitive understanding of the amplitude precision behavior.

### 3.5. Observations

The following figures in this section utilize the parameters mentioned in Table 1, unless mentioned otherwise. Equations (8) and (9) are further explained using the exemplary sine signal. The signal amplitude {*C*} was chosen to match the detected signal amplitude in the experimental results, explained in Section 5.

**Figure 4:**
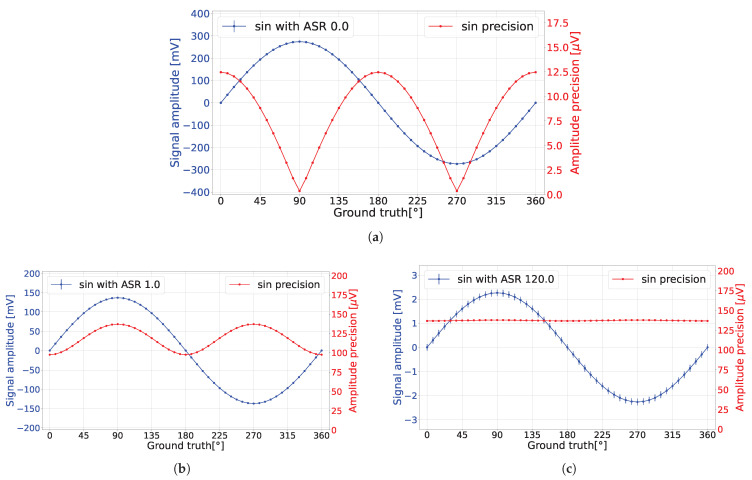
(**a**) When ASR = 0, the analytical model predicted that the detected voltage precision was oscillating due to active light shot noise. (**b**) When ASR = 1, the analytical model predicted that the detected voltage precision was oscillating due to the influence of laser and ambient light shot noise. (**c**) When ASR = 120, the analytical model predicted that the detected voltage precision oscillation was not significant due to the dominant ambient light shot noise.

In the absence of ambient light (ASR = 0), the amplitude precision exhibited oscillation, as demonstrated in Figure 4a. To intuitively understand this phenomenon, recall that the pixel converts the time domain of detected events into the voltage domain via sampling and averaging the corresponding sinusoidal signal with a laser pulse width {*a*}. Consequently, at points where the voltage remains relatively constant over {*a*} (i.e., at the peaks of the sinusoidal signals), the output voltage remains unaffected by laser shot noise. However, at the inflection point at a 180° angle, fluctuations in the detected voltage occurred due to laser shot noise. The transition from the first case to the latter case resulted in oscillation of the amplitude precision.

In the presence of ambient light (ASR = 1), the amplitude precision exhibited a constant shift and an oscillation, as demonstrated in Figure 4b. Due to the ambient light shot noise, a constant amplitude precision offset was imposed on the system. Nonetheless, laser shot noise continued to impact the amplitude precision, with heightened oscillations occurring particularly when the laser aligned with sinusoidal peaks.

To explain this behavior, recall that the resultant output voltage stems from averaging across the entire signal and fluctuations due to both laser and ambient shot noise. Therefore, the voltage fluctuation was influenced by the amplitude difference between the measured voltage amplitude and the remaining sinusoidal signal amplitude. Consider the sine peak point at a 90° angle and the inflection point at a 180° angle. Given that the maximum amplitude difference within the sinusoidal signal was between its peaks, the detected fluctuation attained its maximum when the laser pulse captured the peak voltage of the 90° angle. Conversely, when examining the 180° angle, the discrepancy between the measured voltage amplitude and any peak amplitude was minimized, resulting in the least amount of voltage fluctuation.

Finally, under the condition of elevated ambient light irradiance (ASR = 120), the amplitude precision approached near flatness, as illustrated in Figure 4c. The term corresponding to ambient light shot noise in Equation (8) is dominant, minimizing the impact of laser pulse shot noise.

### 3.6. Phase Calculation

After estimating the expected amplitude and amplitude precision of the sinusoidal signals, the phase information can be extracted via the equation $\theta \left(x,y\right)=\arctan\left(\hat{I}/\hat{Q}\right)$. However, due to the nonlinear relation between the functions, the phase’s expected value and variance are approximated using Taylor expansion. The average phase after the EWMA is

(10) $$\begin{array}{ccc} E[\theta ](l) & \approx & \arctan\left(\frac{E[\hat{I}]}{E[\hat{Q}]}\right)+\frac{E\left[\hat{I}\right]\cdot E\left[\hat{Q}\right]}{{\left(E{\left[\hat{I}\right]}^{2}+E{\left[\hat{Q}\right]}^{2}\right)}^{2}}\left(\sigma_{\hat{Q}}^{2}-\sigma_{\hat{I}}^{2}\right) \end{array}$$

(11) $$\begin{array}{ccc} \sigma_{\theta }^{2}\left(l\right) & \approx & \left(\frac{E{\left[\hat{Q}\right]}^{2}}{{\left(E{\left[\hat{I}\right]}^{2}+E{\left[\hat{Q}\right]}^{2}\right)}^{2}}\sigma_{\hat{I}}^{2}+\frac{E{\left[\hat{I}\right]}^{2}}{{\left(E{\left[\hat{I}\right]}^{2}+E{\left[\hat{Q}\right]}^{2}\right)}^{2}}\sigma_{\hat{Q}}^{2}\right). \end{array}$$

**Figure 5:**
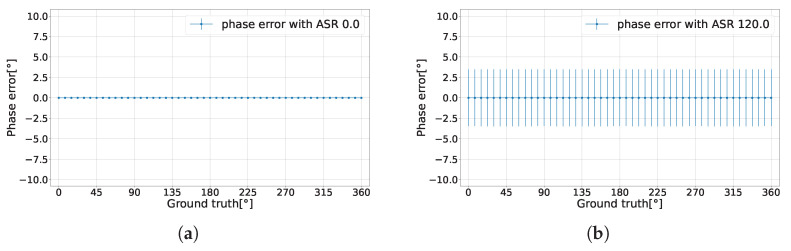
Analytical phase error with the corresponding phase precision. (**a**) When ASR = 0. (**b**) When ASR = 120.

**Figure 6:**
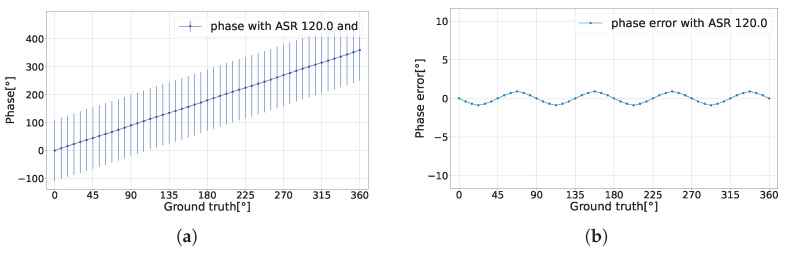
(**a**) Phase’s expected value with the ground truth when ASR = 120 and $n_{av}=1000$. (**b**) Phase error without the corresponding phase precision.

The findings reveal a notable characteristic of the CA-dToF system. For the phase’s expected value E[θ], the variance in the two analog channels due to ambient shot noise was effectively eliminated, leaving only the variance related to the laser pulse width as shown in Figure 5a,b with a phase precision $\sigma_{\theta }$ less than 1% of the detection range when ASR = 120. The difference between the ground truth and the phase expected value is defined as the phase error. The laser pulse width contributes to the oscillation in the phase error in the case of extreme ASR and low integration length $n_{av}$, as shown in Figure 6.

The phase precision was derived from the combination of the analog channel variances. Unlike the phase’s expected value, the phase precision increased, corresponding to the variance in both analog channels. Nevertheless, it was possible to reduce the phase precision by increasing the integration length $n_{av}$.

The analytical model does not account for the effect of the SPAD deadtime. To examine the impact of deadtime and validate the analytical model, a separate statistical model was developed.

## 4. Statistical Model

The pixel is statistically simulated by considering two assumptions: (1) the SPAD generates a digital pulse when triggered, and (2) when the SPAD is triggered, the system evolves via EWMA as presented in Equation (1). The simulation did not explicitly consider the characteristics of the detected object nor the sensor’s dark count rate, as they could be implicitly included in the ASR. The ASR was fixed through the detection range, eliminating the effect of signal reduction for various distances. Ambient light was randomly generated over time, and the laser pulse was a top-hat laser pulse with a fixed pulse width {*a*}. The number of photons over one period followed a Poisson distribution for all events. Two orthogonal sinusoidal signals were sampled simultaneously when the SPAD was triggered, making both signals have the same phase when detected. The parameters used in the simulation are summarized in Table 2. The simulation input parameters were chosen to match the measurement input parameters, as explained in Section 5.

There were multiple simulations conducted to verify the analytical model developed in Section 3 in the absence of the deadtime effect. The focus was on varying the ambient light level and the integration length. The latter allowed avoiding the inertia effect within a limited number of cycles.

### 4.1. Various Ambient Light Conditions

**Figure 7:**
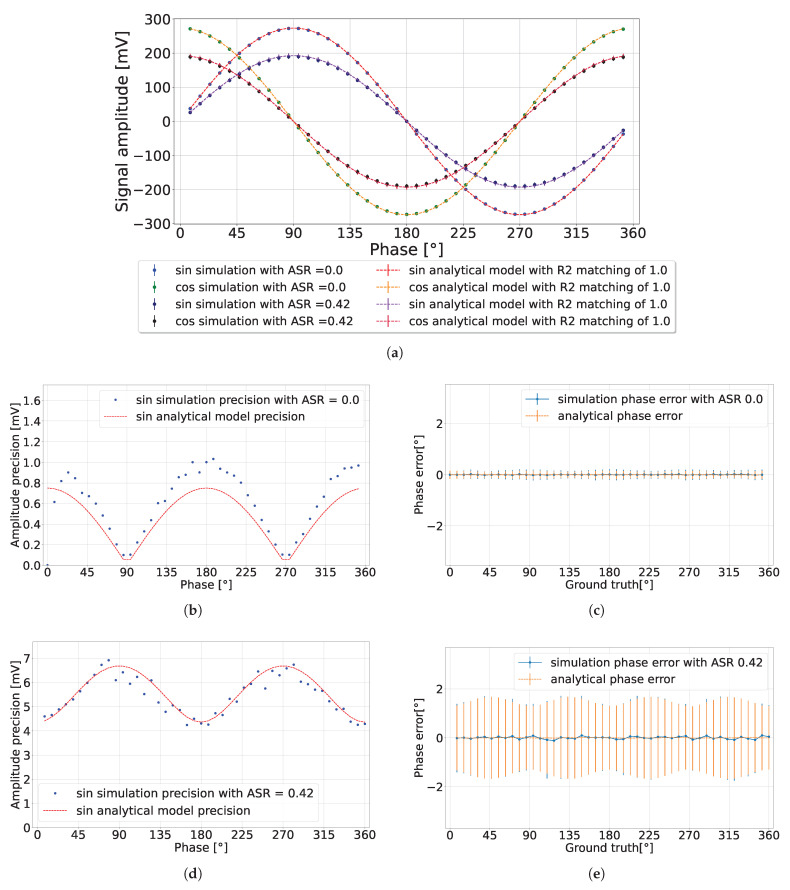
CA-dToF simulation results when $n_{av}=300$ for two different ASR values. (**a**) Detected signal amplitude. (**b**,**c**) Amplitude precision and phase error when ASR = 0, respectively. (**d**,**e**) Amplitude precision and phase error when ASR = 0.42, respectively.

This subsection presents the pixel simulations, comparing them with the developed analytical model presented in Section 3 with the lowest precision tolerance (L = 1). In the simulation results presented in Figure 7, with $n_{av}=300$, each data point was derived from an average of 300 measurements to calculate the detected signal’s expected value and amplitude precision. It can be observed in Figure 7b that in the absence of ambient light (ASR = 0), the amplitude precision exceeded the predicted value by 0.25 mV. This discrepancy may be attributed to our choice of using the lowest precision tolerance of the analytical model (L = 1). Hence, it allowed for the possibility of greater observed amplitude precision. Conversely, when ASR = 0.42, the amplitude precision aligned closely with the predicted analytical model as presented in Figure 7d. The calculated phase error is presented in Figure 7c,e, showing consistent results with the analytical model.

**Figure 8:**
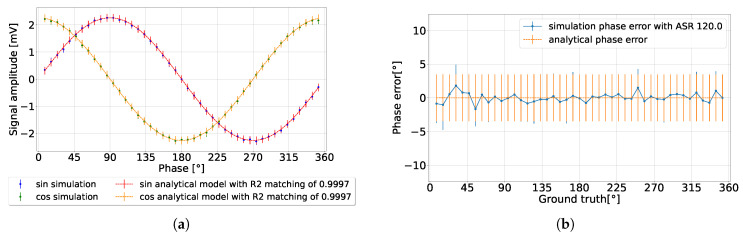
CA-dToF simulation results for $n_{av}=10^{6}$ when ASR = 120. (**a**) Detected signal. (**b**) Phase error.

By changing the environment to a high ambient light setting (ASR = 120 for $n_{av}=10^{6}$), as presented in Figure 8, the detected signal exhibited a significant reduction in amplitude, matching the analytical model results. Concurrently, the amplitude precision was reduced by increasing the integration length, thereby achieving a phase precision that fell below 1% of the detection range, without discrepancy in the phase error. One observation of high ambient light conditions is the diminished detected amplitude, which imposes a constraint on the operational range of the analog-to-digital converter (ADC).

This subsection compared the analytical and statistical results, presenting consistent results between both models. However, the pixel operation was affected by the SPAD deadtime. We explore the deadtime effect on the pixel operation in the following section.

### 4.2. Deadtime Shadowing Effect

**Figure 9:**
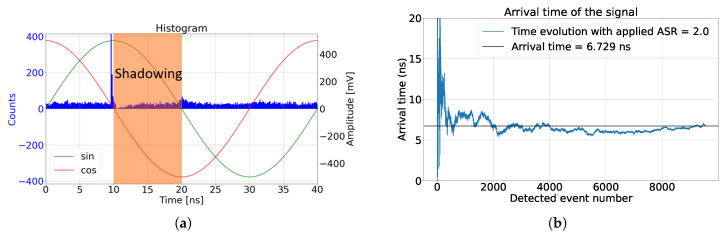
CA-dToF simulation results when ASR = 2 with applied laser pulses arriving at 10 ns and an SPAD deadtime of 10 ns, including a PDP of 0 during the deadtime. (**a**) Detected signal with SPAD deadtime shadowing effect and (**b**) detected phase.

The SPAD deadtime is the time taken for the SPAD to fully recharge into the Geiger operation mode. In our simulation, the SPAD was passively quenched with a resistor. The excess biased voltage of the SPAD charges exponentially, as explained in [19]. If we assumed that the SPAD’s photon detection probability (PDP) was zero during the deadtime, then the detected ambient light was not uniform, as presented in Figure 9. This phenomenon is known as the pile-up effect [17]. However, the operation of the CA-dToF is different than that in the histogram method. Therefore, we refer to this deadtime effect as the deadtime shadowing effect, where the SPAD deadtime shadows the rest of the active signal and the detected ambient light, making the ambient light nonuniform over the applied signal and creating a phase offset which depends on the ambient light level. In the remainder of this section, an investigation is conducted to predict the deadtime shadowing effect over CA-dToF’s operation.

**Figure 10:**
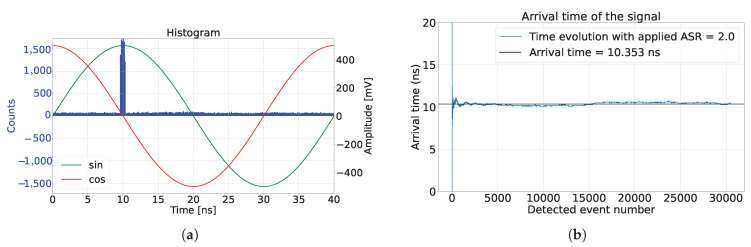
CA-dToF simulation results, including a PDP relative to the SPAD excess bias during the deadtime. (**a**) Detected signal and (**b**) detected phase.

The assumption that the PDP is zero can be challenged when using a passively quenched SPAD. As mentioned in [20], the SPAD PDP can be approximated to have a linear response with the excess bias. Hence, by considering the increase in the SPAD PDP with the exponential increase in the excess bias, we noticed that the shadowing effect was significantly reduced, as illustrated in Figure 10. One conclusion drawn from this analysis is that the deadtime shadowing effect can be nulled by a sufficient number of photons in the detected laser pulses.

**Figure 11:**
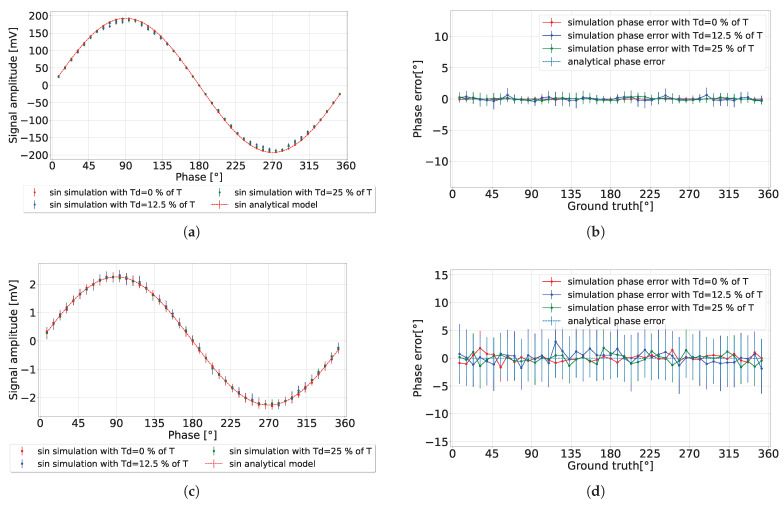
CA-dToF simulation results for two different SPAD deadtime values for $n_{av}=300$ when ASR = 0.42. (**a**) Detected sine signal and (**b**) phase error. When ASR = 120 and $n_{av}=10^{6}$, (**c**) is the detected sine signal, and (**d**) is the phase error.

To investigate further, we implemented a passively quenched SPAD with different deadtime values to our simulator, neglecting the voltage threshold of the detection circuit after the SPAD. The results are presented in Figure 11. We scanned the full range of detection when ASR = 0.42 and $n_{av}=300$, as presented in Figure 11a,b. The same scenario was also simulated when ASR = 120 and $n_{av}=10^{6}$, as depicted in Figure 11c,d. The simulation showed that the shadowing effect did not impact the phase error with different deadtime values or different environmental conditions.

Mitigation of the shadowing effect can be achieved through the employment of multiple SPADs per pixel. In this configuration, each SPAD is coupled with a switched capacitor circuit, and the resultant signal output is integrated within a larger switched capacitor. This approach serves to further alleviate the shadowing effect by enabling the independent triggering of multiple SPADs.

Additionally, an alternative method for mitigating the shadowing effect involves the utilization of SPAD active quenching synchronized with the signal period {*T*}. This strategy effectively eliminates the shadowing effect by ensuring that the detection occurs only once within each signal period. This solution relies on the fact that when one photon is detected per period, the ambient light detection becomes uniform, thus facilitating ambient light averaging and subsequent mitigation of the shadowing effect. Conversely, the system would require a higher number of cycles to reach equilibrium, consuming more power on average and having a lower frame rate. The shadowing effect was not observed when our experimental work was conducted, as presented in Section 5.

## 5. Experimental Results

### 5.1. Experimental Set-Up

**Figure 12:**
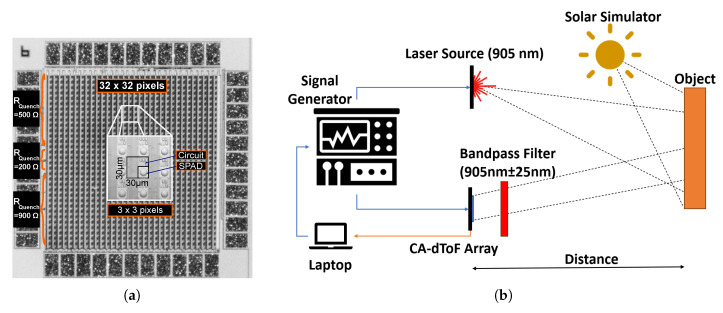
(**a**) CA-dToF pixel array micrograph with three different quenching resistors. (**b**) The experimental set-up.

Following the statistical analysis of the system operation, experimental work was conducted. A pixel array schematic, presented in Figure 1a, was fabricated using X-FAB 180 nm technology with a pixel pitch of 30 μm, as presented in Figure 12a. The sinusoidal signals were synchronized with a 905 nm high-power pulsed laser source with a top-hat pulse width of 1.8 ns using a signal generator. Sinusoidal signals with a 25 MHz repetition rate were applied to the full array with $n_{av}\approx 300$ per analog channel. The pixel array featured an identical circuit structure across the array, except for the quenching resistor $R_{Quench}$. Three different values for the quenching resistors were used—200 kΩ, 500 kΩ, and 900 kΩ—as presented in Figure 12a. The quenching resistor variations were meant to test the deadtime variation effect over the pixel operation. The exact deadtime was not accessible to be detected. However, the deadtime was expected to be higher for a higher-resistance quenching resistor.

The experimental set-up is presented in Figure 12b, with a solar simulator device (Hal-302) utilized as the ambient light source to emulate sunlight.

### 5.2. Measurement Method

To align the analytical and statistical models with the experimental work, the ASR had to remain constant across the detection range. Therefore, the distance between the CA-dToF array and the object was fixed, thereby preventing ASR degradation over varying distances. Object movement was mimicked by shifting the applied sinusoidal signal using the signal generator, which scanned the entire detection range. Additionally, we measured the circuit behavior across the full detection range at different ASR levels. The applied sinusoidal signals had a peak-to-peak voltage of 1.20 V. As shown in Figure 1a, the voltages of the switched capacitor stages (SC1 and SC2) were detected through two PMOS source followers ($M_{5}$ and $M_{6}$).

To account for the source follower offset of the sinusoidal signal $V_{offset}$, a differential measurement was employed. In this method, two detected measurements were shifted 180°, which was achieved by shifting either the applied laser or the applied signal 180°. For example, for the sine signal, the detected voltages were $\left(\hat{C}\sin\left(\theta \right)+V_{offset}\right)$ and $\left(\hat{C}\left(-\sin\left(\theta \right)\right)+V_{offset}\right)$, where $\hat{C}$ represents the detected amplitude of the sinusoidal signal. By averaging the two measurements, the offset voltage $V_{offset}$ was determined. Another method for detecting the voltage offset is by measuring ambient light only without any laser signal. In this case, the average voltage should theoretically reach zero, indicating the offset of the source follower, as discussed in Section 3.1. However, this method relies on the assumption that the system’s switched capacitor and the source follower responses are linear across the entire detection range. Observations indicated that this linearity was not maintained over the full range of detection, leading to the decision to not use this method in our measurements.

The experiment was conducted using the set-up illustrated in Figure 12b. Both the ambient light and laser source were projected onto an object with an approximate reflectivity coefficient of 24% for the wavelength used. The object remained at a constant distance throughout the detection period. To prevent SPAD saturation, the ambient light was filtered using a bandpass filter centered near a 905 nm wavelength. Each distance was measured 1024 times to determine the signal amplitude mean and amplitude precision associated with each point. This data analysis aimed to identify the system’s minimum amplitude precision to align the results with the analytical model. Consequently, the control width *L* in Equations (8) and (9) was set to one. The used SPAD had a PDP of 5% for a 905 nm wavelength.

### 5.3. Experimental Results

**Figure 13:**
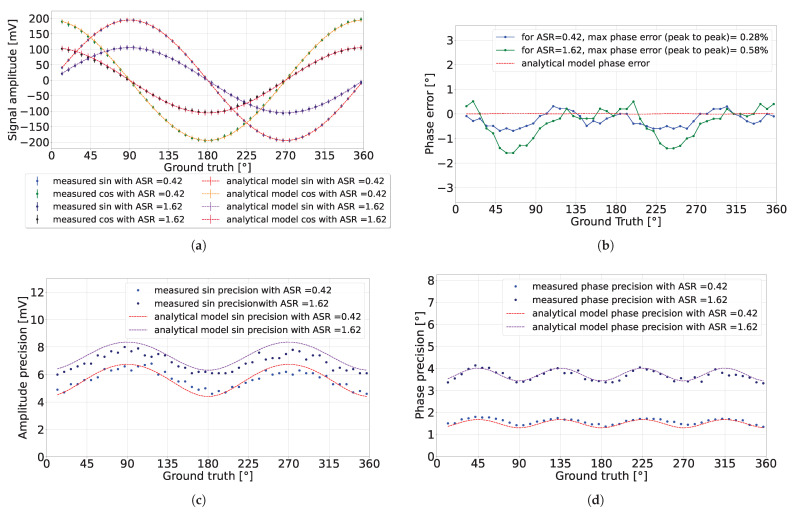
CA-dToF pixel experimental results for two different ASR values: (**a**) detected signal, (**b**) detected phase error, (**c**) detected amplitude precision, and (**d**) detected phase precision.

The detected signal over the full phase range is presented in Figure 13, with ASR values of 0.42 and 1.61. Figure 13a shows the detected sinusoidal amplitude relative to the ground truth in the phase, with a phase step delay of 7.5° applied by the signal generator. The ground truth is expressed in terms of its phase rather than time to generalize the measurement for testing the pixel operation independent of the applied signal period. The analytical model with the same parameters is also plotted in the figure. The pixel used for this result had an SPAD quenching resistor of 500 kΩ.

The detected sinusoidal signals exhibited different amplitudes at 20 mV, possibly due to a gain mismatch between the source followers of the two analog channels. After calibration, the maximum deviation between the analytical model and the measurements was near 8 mV. This difference in sinusoidal amplitudes translated into cyclic phase error, as shown in Figure 13b.

The precision in the detected sine amplitude, shown in Figure 13c, matched the behavior predicted by the analytical model, with a similar pattern observed for the detected cosine amplitude precision. The amplitude precision in the detected sinusoidal signals translated into the phase precision, illustrated in Figure 13d, which also aligned with the predictions of the analytical model.

**Figure 14:**
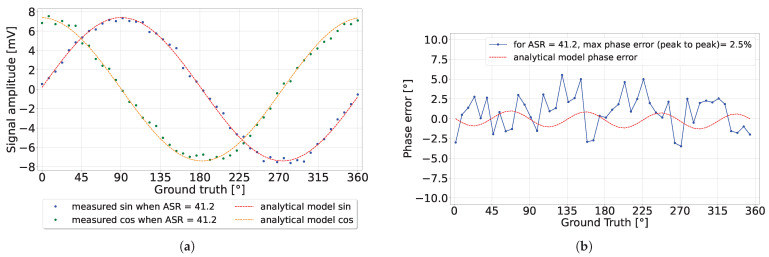
The detected sinusoidal amplitude (**a**) and phase error (**b**) when ASR = 41.2.

Under a high ASR of 41.2, the detected signal amplitude approximately matched the analytical model. However, the overwhelming ambient light shot noise affected the detected phase, causing the phase error to oscillate, as shown in Figure 14b. The phase precision was omitted from this figure due to its high value of approximately 90°. Overall, the results between the analytical model and the measurements consistently matched.

The pixels consumed 40 μW per pixel on average, including the SPAD operation, two switched capacitor circuits, and an in-pixel counter. The power consumption of the supporting systems, such as the laser source and the generated signals, was not considered, as we were focused on characterizing the pixel operation.

**Figure 15:**
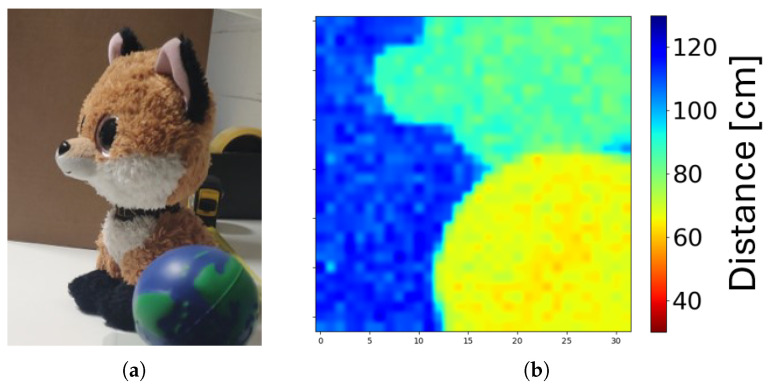
A snapshot of a scene with the 32×32 pixel array at the room’s ambient light. (**a**) Colored image of the scene. (**b**) The 3D image.

To characterize the SPAD deadtime shadowing effect, two pixels with 200 kΩ and 900 kΩ were tested at a fixed detected distance. If there was a deadtime shadowing effect, then we expected phase degradation with different ambient light conditions for different deadtime values, leading to a distance error. Table 3 presents two pixels with different quenching resistors, detecting the same distance with two different ASR conditions. The maximum distance error detected was 0.3% for a 6 m detection range. The phase error did not present reliable evidence of the shadowing effect. In Figure 15, a 3D image and a colored image were captured by the pixel array over a 1 m range.

Table 4 presents a comparison between our pixel operation and different dToF pixels. The key criterion was the pixel power consumption, which indicates the scalability of a pixel array. Pixel precision was not considered, as the presented pixel was limited by its internal integration length. The primary source of power consumption in the CA-dToF pixel is the SPAD sensor, with the pixel circuit consuming four times less power compared with the SPAD.

## 6. Conclusions and Discussion

This paper introduced the operation of a correlation-assisted direct time-of-flight (CA-dToF) imaging sensor which utilizes an SPAD-based pixel array and sinusoidal signals correlated with a pulsed laser source. The pixel operation was comprehensively analyzed using an analytical model and validated through statistical modeling and experimental work. The pixel operation conceptually depends on the laser pulse width, the ASR, the sinusoidal signal period and amplitude, and the integration length of the pixel.

The operational range of the pixel depended on the applied sinusoidal signal period. However, the detected precision decreased over longer ranges. Another challenge was phase wrapping in the detected signal, which led to distance detection ambiguity. To maintain high-precision detection and avoid phase wrapping, gating the sinusoidal signal is a viable solution. This method involves initially applying a frame with a long period {*T*} to estimate the approximate distance of the object. Subsequently, a second frame with a gated sinusoidal signal of a period {*T*}/*n*, where *n* is an integer, is applied to the pixel, being gated around the object’s approximate distance. The first frame provides a rough estimate of the object’s distance, while the second frame offers higher precision and a more accurate distance measurement.

The pixel operation did not directly account for environmental effects. The reflectivity of the detected object and its location affected the ASR of the detected signal. The frame rate of the pixel depended on the integration length, the detected environment, and the SPAD sensitivity to the detected wavelength. The analytical model in Section 3 was based on the assumption that the pixel-detected voltages reached equilibrium. However, for a high integration length value $n_{av}$, a high frame rate, and a low ambient light level, the inertia effect, discussed in Section 2, may lead to inaccurately detected voltages, causing a mismatch between the analytical model and the measurement. Under a low light condition and a high frame rate, the inertia effect can cause errors in the detected confidence and phase. To overcome this challenge, one possible solution is to dedicate testing pixels with a low integration length, which reach equilibrium faster, and to use their data for checking if the pixels reach equilibrium. Another suggestion is to develop variable capacitors in-pixel to adjust the pixel integration length, depending on the environmental conditions. However, this solution requires an extra system to detect the outside environment’s photon flux.

Additionally, the SPAD deadtime is not considered in the analytical model. We address in Section 4.2 the SPAD deadtime shadowing effect on the phase and propose possible mitigation methods. Other environmental conditions which affect ToF technologies such as the scattering environment, multi-path reflections, and transparent objects are also challenges to the proposed CA-dToF pixel. More studies are required to understand the influence of such challenges on CA-dToF pixel operation.

## Figures and Tables

**Table 1:** Parameter values used in the remainder of this section.

Parameter	Value
Pulse width {*a*}	4.25% of signal period
Signal amplitude {*C*}	274.6 mV
Integration length {$n_{av}$}	$10^{6}$

**Table 2:** The parameters used for the statistical model.

Parameter	Value
Pulse width {*a*}	4.25% of signal period
Signal amplitude {*C*}	274.6 mV
Number of cycles	1000

**Table 3:** A fixed distance point was analyzed over $10^{5}$ data samples to detect the shadowing effect over the detected distance. The ground truth is the distance when ASR = 0, and the error is the difference between the ground truth and the detected distance when the ASR is high for the same pixel, divided by the full detection range of 6 m.

Quenching Resistor	Condition	Distance (cm)	FL Distance Precision (cm)	FL Distance Error (%)
200 kΩ	ASR = 0	555.03	0.57	0.30
	ASR = 61	553.22	8.36	
900 kΩ	ASR = 0	554.34	0.54	−0.08
	ASR = 55	554.83	6.77	

**Table 4:** Comparison of performance with different dToF pixels.

Parameter	Unit	This Work	[21]	[22]	[23]
Year	-	2024	2019	2022	2021
Technology	nm	180	180	65/65	90/40
		FSI	FSI	3D-BSI	3D-BSI
Pixel array	-	32 × 32	252 × 114	240 × 160	320 × 240
Pixel pitch	μm	30	28.5	16	12.5
Power per pixel	μW	40	70	600	300
Maximum distance error ${\;}^{1}$	%	0.28	0.17	0.1	<5
Detection range	m	6	50	9.5	6
Pulse width	ns	1.7	0.04	-	0.36
Wavelength	nm	905	637	940	940

${\;}^{1}$ For low ambient light conditions.

**Figure A1:**
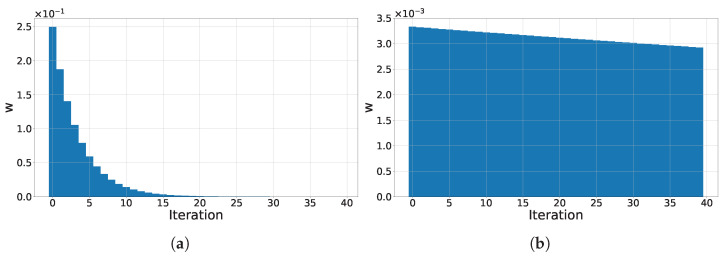
Measurement weight evolution when (**a**) λ=0.25 and (**b**) $\lambda =\frac{1}{300}$. The blue bar indicates the effect of the new measurement on the previous measurement weight.

## Data Availability

Data are contained within the article.

## Abbreviations

The following abbreviations are used in this manuscript:

EWMA	Exponential weighted moving average
CA-dToF	Correlation-assisted direct time-of-flight
ASR	Ambient-to-signal ratio
PDP	Photon detection probability

## Appendix A. Exponential Weighted Moving Average

Exponential moving average, geometrical moving average, and exponential weighted moving average (EWMA) are various names for the same averaging method. It is an average where the previous measurements are exponentially decrementing their weight over new iterations. To observe this behavior, the general equation is

(A1) $$V_{i}=x_{i}\lambda +\left(1-\lambda \right)V_{i-1},$$

as 0<λ<1, which is the weight. To understand the behavior of λ, let us observe the evolution of the equation:

(A2) $$\begin{array}{ccc} V_{i} & = & \lambda x_{i}+\left(1-\lambda \right)V_{i-1}=x_{i}\lambda +\lambda \left(1-\lambda \right)x_{i-1}+{\left(1-\lambda \right)}^{2}V_{i-2}\\ & = & \lambda \sum_{j=0}^{i-1}{\left(1-\lambda \right)}^{j}x_{i-j}+{\left(1-\lambda \right)}^{i}V_{0} \end{array}$$

With Equation (A2), we can define the weighted factor $w_{j}$ as $w_{j}=\lambda {\left(1-\lambda \right)}^{j}.$

### Appendix A.1. Properties

The first observation regarding $w_{j}$ is its convergence to one over infinite iterations, as calculated below:

(A3) $$\begin{array}{ccc} \sum_{j=0}^{i}w_{j} & = & \lambda \sum_{j=0}^{i}{\left(1-\lambda \right)}^{j}\\ & = & \lambda \frac{1-{\left(1-\lambda \right)}^{i}}{1-(1-\lambda )}=1-{\left(1-\lambda \right)}^{i}, \end{array}$$

where the term $\sum_{j=0}^{i-1}{\left(1-\lambda \right)}^{j}$ is a geometric series. The observation implies that if there is a constant voltage $V_{0}$ measured over infinite iterations, then the output voltage $V_{i}$ converges to $V_{0}$. For λ=0.25 and $\lambda =\frac{1}{300}$, the weighted factor $w_{j}$ is dwindling as shown in Figure A1a,b, respectively.

An important observation here is that for a smaller λ, the decay of $w_{j}$ is slower over new iterations. Hence, for small λ values, the EWMA can be used to approximate the usual arithmetic mean for a certain detection period. But when mentioning the detection period, we assume that the rate of detection is fast enough that the system reaches equilibrium. If the detection rate is insufficient, then the system cannot converge to the average value. The inertia effect is further discussed in Appendix A.3.

### Appendix A.2. EWMA Standard Deviation

If a system generates random and uncorrelated measurements with a variance $\sigma^{2}$, then the variance of the EWMA $V_{i}$ is reduced as shown in the following the equation [18]:

(A4) $$\sigma_{i}^{2}=\sigma^{2}\left(\frac{\lambda }{2-\lambda }\right)\left[1-{\left(1-\lambda \right)}^{2i}\right].$$

For an infinite number of iterations, the variance converges to a steady state:

(A5) $$\sigma_{i}^{2}=\sigma^{2}\left(\frac{L^{2}}{2n_{av}-1}\right)\;1\leq L\leq 3,$$

where $n_{av}=1/\lambda$ and *L* is the control width, which represents the tolerance of the model at which the system is still under control and is tuned by the system data.

This result implies that (1) the variance of the system is filtered out via the EWMA process, and (2) the smaller the λ value, the lower the variance output of the system. These conclusions are interesting, as there is control over the variance of the system by controlling the $n_{av}$ value.

### Appendix A.3. Inertia Effect

To have a better understanding of the weight factor $w_{j}$, we sum the weight of a measurement $x_{i}$ after *N* iterations. Using Equation (A3), we can find that

(A6) $$\begin{array}{ccc} \gamma_{N} & = & \sum_{i=N}^{\infty }w_{i}=\sum_{i=0}^{\infty }w_{i}-\sum_{i=0}^{N}w_{i}\\ & = & 1-(1-{\left(1-\lambda \right)}^{N})\\ & = & {\left(1-\lambda \right)}^{N}\;0\leq \gamma_{N}<1 \end{array}$$

Equation (A6) provides an important relationship between the number of iterations *N* and the weight evolution $\gamma_{N}$. For example, to have 99% of the measurement weight, the neglected weight is $\gamma_{N}=1\%$. The number of iterations to reach a certain lower weight $\gamma_{N}$ is calculated to be

(A7) $$N=\frac{log(\gamma_{N})}{log(1-\lambda )}$$

For example, let $\lambda =\frac{1}{300}$ with an initial weight measurement $w_{0}=0.0033$. For this measurement weight to reach ≈70% of the initial weight, the neglected weight is $\gamma_{N}\approx 30\%$. The number of iterations is $N=\frac{log(0.3)}{log(1-\frac{1}{300})}\approx 361$, with a weight $w_{361}\approx 0.0010$. This result implies that with a smaller λ value, the initial weight decay is slower, enabling better averaging. However, the system’s response to a sudden change is poor for a small λ value. It requires multiple iterations to react to the change in the measurement, meaning that the EWMA system has “*inertia*” against new changes in the system. This inertia effect reduces the effectiveness of adapting to a new change in the system.
